# The influence of learning styles on academic procrastination among students in mathematics

**DOI:** 10.3389/fpsyg.2023.1239933

**Published:** 2023-10-26

**Authors:** Wan Anis Syamimi Wan Hussin, Mohd Effendi Ewan Mohd Matore

**Affiliations:** ^1^Faculty of Education, The National University of Malaysia (UKM), Bangi, Selangor, Malaysia; ^2^Research Centre of Education, Leadership and Policy, Faculty of Education, The National University of Malaysia (UKM), Bangi, Selangor, Malaysia; ^3^University Research Groups (KPU), Educational Evaluation, The National University of Malaysia (UKM), Bangi, Selangor, Malaysia

**Keywords:** learning styles, academic procrastination, VAK modality, mathematical concepts, secondary school students

## Abstract

**Introduction:**

Procrastination is a complex psychological and behavioral construct that is strongly influenced by certain personality traits. In mathematics learning, students find it difficult to master the concepts because of less exposure to learning styles. Poor knowledge of mathematical concepts leads to academic procrastination in the subject of Mathematics among students. Therefore, this study aims to identify students’ learning styles in Mathematics, identify the stages of students’ academic procrastination in Mathematics, and determine whether there is a significant influence of learning styles (visual, auditory, and kinesthetic) on academic procrastination among secondary school students in Mathematics.

**Methods:**

A quantitative approach with a survey was applied. A total of 500 Form Two and Form Four students in five national secondary schools in the Kota Bharu district, Kelantan, were selected using simple random sampling. The duration of data gathering started from 4 October 2022 until 31 January 2023. The Learning Styles Questionnaire and the Academic Procrastination Questionnaire were adapted and verified by eight experts in psychology and counseling. Descriptive and multiple regression tests were carried out using IBM SPSS version 26.0.

**Results:**

The results revealed that the visual learning style was the most dominant learning style among students in the subject of Mathematics, followed by auditory and kinesthetic. The level of students’ academic procrastination in Mathematics was low. Besides, multiple regression showed that visual and kinesthetic learning styles were significant contributors or predictors, which amounted to 14.1% of the variation in students’ academic procrastination in Mathematics.

**Discussion:**

The implications of this study highlight the possibility to improve programs in schools by exposing students to suitable learning styles so that they can practice effective learning styles in Mathematics and consequently overcome academic procrastination. Further research can be carried out by identifying other factors that encourage academic procrastination in the subject of Mathematics in order to increase students’ motivation and self-efficacy.

## 1. Introduction

Good cognitive skills are essential in mathematics learning so that students can understand and master mathematical concepts. Different learning styles are identified in mathematics learning. There are three types of learning styles that students often practice in the subject of Mathematics, namely visual, auditory, and kinesthetic (VAK) learning styles (Kurniawan and Hartono, 2020). This is because VAK learning styles are more famous and easy to use in identifying one’s learning style (Febriani, 2018; Zulkipli et al., 2019). Thus, appropriate learning styles in the subject of Mathematics help students to understand mathematical concepts more effectively (Cimermanová, 2018), in addition to creating a more fun mathematics learning atmosphere since it is more interactive (Osman et al., 2019). Study by Asio (2020) found that respondents tend to procrastinate with their academics. There were significant differences reported when the respondents’ academic procrastination response is grouped according to the program, scholarship status, and religion. This means that the difference in academic procrastination requires further study to identify the cause.

Characteristics of students’ learning styles can be identified during mathematics learning. Students with a visual learning style learn mathematical concepts through images such as by depicting formulas (Machromah et al., 2021). For example, in the topic of geometry, students with a visual learning style analyze geometric shapes through pictures. Meanwhile, students with an auditory learning style listen to the teacher’s explanation of the mathematical topic being studied (Rahman and Ahmar, 2017), and they always repeat mathematical concepts such as saying mathematical formulas regularly so that they can remember the formulas better. Finally, students who practice a kinesthetic learning style often apply mathematical topics to everyday life (Irvine, 2019). For example, in algebra topics, students are given real situations to form algebraic expressions (Indraswari et al., 2018). Therefore, with an effective learning style, students can master mathematical concepts more quickly and easily.

When students can understand the concepts and content of mathematics effectively, they can complete the tasks given by the teacher excellently without delaying them (Fulano et al., 2021). However, due to the lack of understanding of difficult mathematical concepts, students are not interested in mathematical tasks (Gonda et al., 2021) because they can neither re-explain the concepts nor apply those concepts to solve mathematical problems. Students also find it difficult to relate newly learned mathematical concepts to other mathematical concepts (Ulum and Pujiastuti, 2020).

Most students only memorize formulas and steps to solve mathematical problems without understanding them. This causes students to experience difficulties in solving questions or other mathematical problem-solving exercises even if they involve the same question form (Yuanita et al., 2018). Students also think that mathematics assignments are too complicated and difficult because mathematics learning is more abstract (Hui and Rosli, 2021). Therefore, practicing the right learning style can create a positive atmosphere when learning mathematics (Mundia and Metussin, 2019) and subsequently reduce the tendency of students to practice academic procrastination in the subject of Mathematics. Moreover, academic procrastination in the subject of Mathematics also occurs due to a lack of self-confidence and possible fear of the subject (Agustin and Winarso, 2021).

Based on a study of academic procrastination involving students in Indonesia, the late submission of Mathematics assignments was the most prominent (44%), followed by Physics (31%), and other subjects (28%; Setiyowati et al., 2020). Mathematics education in Malaysia involves many exercises to strengthen students’ understanding of mathematical concepts (Sarudin et al., 2022). Teachers usually give assignments or mathematical exercises in textbooks or reference books as homework so that problem-solving skills and mathematical theories and concepts can be honed and improved (Azhar and Rosli, 2021). These mathematics assignments or exercises will then be discussed in the next class, and students may also need to complete them before class. Hence, the suitability of students’ learning styles plays a role in encouraging students to complete mathematics assignments.

One of the reasons for less effective learning styles is that students are not given enough exposure to learning styles at an early stage (Cholifah et al., 2018; Ismajli and Imami-Morina, 2018). This is because students are not given specific guidance on the way or style of learning. A less effective learning style causes students to be less interested in the subject of Mathematics and this will lead to academic procrastination because students do not have sufficient basic knowledge of Mathematics (Fulano et al., 2021). Differences in the learning style of each student require teachers to identify and adapt mathematics teaching methods to students. One of the factors influencing students’ inability to master the learning content well is the teacher’s teaching style, which is not a match to the variety of learning styles that students have (Mustaffa et al., 2021). Thus, teachers need to identify students’ learning style patterns in order to better plan teaching and learning strategies based on the characteristics of each learning style (Zulkipli et al., 2019; Abd Shukor and Masroom, 2020).

Studies have found that many teachers still prioritize the conventional method of teaching Mathematics by giving explanations in class (Voon and Amran, 2021). This teaching method is very suitable for students who practice visual and auditory learning styles. This is because students with an auditory modality receive information through hearing, such as discussions and explanations of mathematical concepts. Meanwhile, students with a visual learning style are more likely to learn through sight by looking at the symbols, graphs, or mathematical solutions shown by the teacher on the blackboard (Zulkipli et al., 2019). However, this is quite difficult for kinesthetic students who need hands-on or objects to manipulate in order to understand abstract mathematical concepts that are difficult to explain (Nurrahmah et al., 2021). As such, visual and auditory students may be able to understand mathematical concepts better than kinesthetic students (Umam and Azhar, 2019).

In addition, past studies have also focused more on the relationship between learning styles and other factors such as motivation (Abd Shukor and Masroom, 2020; Noervadila, 2020; Mohamed and Hassan, 2021) and mathematics achievement (Schulze and Bosman, 2018; Altun and Serin, 2019), while studies on the relationship between learning styles and academic procrastination are still limited. Studies on learning styles against academic procrastination should be given attention because students need to identify the learning style patterns to be applied when learning Mathematics. There are previous studies examining the relationship between academic procrastination and student self-regulation in Malaysia (Jerry et al., 2021) as well as the relationship between students’ learning styles and academic procrastination in Istanbul (Gunduz, 2020). However, these two studies do not focus on the context of Mathematics education. Studies related to learning styles and academic procrastination are also reportedly limited and have not been widely studied (Çakır et al., 2014; Gunduz, 2020).

There are several important areas where this study makes an original contribution to identify the aspect of learning styles that are able to influence students’ academic procrastination in Mathematics. Secondly, this contributes to the body of knowledge by expanding the academic procrastination replications onto Mathematics. Besides, these results can benefit schools by developing the training module for students to manage time properly to avoid procrastination. For teachers, this can help them to modify the pedagogy and assessment in class to match with procrastination problems. Besides, parents also can learn how to train their children’s by getting bigger understanding on procrastination.

Therefore, in order to improve the understanding of mathematical concepts among students, this study was conducted to address the following objectives:

1. To identify students’ learning styles in the subject of Mathematics in secondary schools.
2. To identify the level of students’ academic procrastination in the subject of Mathematics.
3. To determine whether there is a significant influence of learning styles (visual, auditory, kinesthetic) on the academic procrastination of secondary school students in the subject of Mathematics.

## 2. Literature review

Learning styles refer to how students acquire knowledge and skills as well as respond to the environment in order to process and interpret information (El-Bishouty et al., 2019; Ahmad and Ambotang, 2020; Shamsuddin and Kaur, 2020). Learning styles also refer to how students focus, store, and process new knowledge (Mohd Zamri et al., 2022). Knowledge can be processed when students plan their learning system such as how to revise lessons and how to complete assignments (Mohamed and Hassan, 2021). Students’ learning styles differ according to their respective inclinations such as visual, auditory, and kinesthetic. Therefore, in this study, learning styles refer to the inclinations of students in processing mathematical knowledge through visual, auditory, or kinesthetic learning styles.

Apipah (2018) stated that visual, auditory, and kinesthetic student characteristics can be identified in mathematics learning. Students with a visual learning style record the steps to solve mathematical problems in a systematic and clear manner. They also think a lot by describing solutions before solving mathematical problems (Machromah et al., 2021). Meanwhile, students with an auditory learning style are also able to write steps to solve mathematical problems. However, they find it difficult to write complete or detailed solution steps. Finally, students with a kinesthetic learning style are more inclined to movements or touch such as holding objects or making body movements when learning mathematics (Irvine, 2019), causing them to be less careful in solving mathematical problems.

Several studies have examined the most dominant student learning style in the subject of Mathematics. For instance, a study by Virgana (2019) found that most students adopted an auditory learning style in mathematics learning, followed by kinesthetic and visual learning styles. However, a study by Nithya Dewi et al. (2019) showed that students with a visual learning style were more dominant, while the kinesthetic learning style recorded the lowest percentage in the subject of Mathematics. Additionally, based on research by Jurenka et al. (2018), the auditory learning style showed the highest percentage compared to kinesthetic and visual learning styles. Evidently, these three different research findings have shown that students tend to adopt a learning style according to their suitability and ability in mathematics learning (Ahmad Damanhuri et al., 2020).

One of the main mathematical learning skills includes making mathematical connections. This skill helps students to connect mathematical ideas in order to formulate knowledge between topics so that mathematical problem-solving can be performed (Savitri and Rochmad., 2022). Indeed, difficulty in understanding the relevance of Mathematics is influenced by learning styles (Apipah, 2018). This is because learning styles typically determine how students develop mathematical knowledge.

Through learning styles, students learn to absorb, organize, and process mathematical knowledge. A study by Agnes and Yunis (2021), which compared visual, auditory, and kinesthetic students in making connections between social arithmetic and addition operations, showed that kinesthetic and visual students were able to connect mathematical concepts well. This study is different from those conducted by Apipah (2018) and Savitri and Rochmad. (2022) in which kinesthetic students were found to lack the skills to connect mathematical concepts. Nonetheless, it should be noted that all three studies found that visual students were able to make mathematical connections excellently.

In addition, Jasmi and Sulaiman (2018) found that students lacked knowledge about their learning styles, making it difficult for them to determine the learning style that suits them and apply an effective learning style in accordance with the mathematics learning process. As a result, students become lazy and less interested in learning Mathematics in class. Problems also occur when students practice a learning style that is not compatible with the teacher’s teaching method (Ismajli and Imami-Morina, 2018). Teachers who tend to explain mathematical concepts without holding activities or discussions in class cause students with different learning styles to pay less attention. When students find it difficult to concentrate during mathematics learning, they will be easily compelled to not complete mathematics assignments as instructed by the teacher and this consequently leads to academic procrastination.

Academic procrastination in Mathematics refers to the act of postponing or delaying mathematics tasks such as delaying mathematical exercises and doing other activities that are more enjoyable (Zacks and Hen, 2018). At school, teachers are not only focused on explaining the concepts and content of mathematics lessons but also on giving assignments in the form of exercises or homework to students to complete at home to hone their mathematical skills. Usually, the assignments or exercises given are taken from mathematics textbooks or exercise books and examples of past questions (Lim and Rosli, 2021). The exercises given are also sometimes considered a form of revision for the topics taught in class to improve the students’ memory of a mathematical topic and familiarize students with mathematical problem-solving. These mathematics exercises are then discussed the next day at school.

Some students were also found to intentionally or unintentionally fail to complete the assignments given by their teachers. Surprisingly, academic procrastination in the subject of Mathematics is considered normal among school students. Previous studies have identified two types of academic procrastination, i.e., sending assignments beyond the cut-off time and delaying the completion of assignments (Rabin et al., 2021). Procrastination in academic assignments is caused by a lack of motivation and the existence of various negative emotions (Rahimi and Vallerand, 2021).

Students who experience anxiety in Mathematics are the cause of increased academic procrastination in Mathematics among students (Paechter et al., 2017). In addition to psychological factors, factors such as lack of understanding and basic knowledge of mathematical concepts also cause students to delay their mathematics assignments. This is because learning involves students’ existing knowledge; therefore, students need to connect it with new information. In this regard, students who have complete basic knowledge understand mathematics lessons better, and this is evident when students complete the mathematics tasks given by their teachers excellently (Fulano et al., 2021).

Studies have also shown that students tend to procrastinate on mathematics assignments because they do not deem the assignments important. For instance, a study by Asri et al. (2017) found that students were less aware of the importance of mathematics assignments given by their teachers; they would only finish the assignments a few minutes before the class starts, and some students would even copy their friends’ steps and answers. Based on data from interviews with several Mathematics teachers, it was found that students were not serious about completing mathematics assignments and would only carry out the assignments for the purpose of fulfilling the teachers’ instructions.

In addition, teachers who are less strict with the submission of mathematics assignments may also cause students to not take the assignments seriously (Nurhadi, 2018). This is because students are not subject to any fines or penalties for submitting their assignments late. In fact, some teachers are only aware that students have submitted their assignments, but they have no idea whether students perform their assignments as soon as given or at the final time of the assignment submission (Asri et al., 2017).

Academic procrastination can affect students’ mental health, as evidenced in a study by Maria-Ioanna and Patra (2022). Therefore, intervention in helping students deal with academic procrastination in learning is very necessary to help students have good mental and physical health so that their learning process is not interrupted. Previous studies by Dewi et al. (2015) and Toker and Avcı (2015) also emphasized the importance of developing cognitive, emotional, and behavioral skills in dealing with academic procrastination. Academic procrastination affects the emotional, behavioral, and cognitive aspects of students, and its impact on students’ emotions can be seen when students find it difficult to initiate, maintain, or complete assignments on time. Procrastination from a behavioral aspect can be translated as a tendency to delay the completion of tasks, while procrastination from a cognitive aspect is seen as the highest level of procrastination. Repetitive procrastination in the emotional, behavioral, and cognitive dimensions is also seen to occur frequently due to the way individuals think.

Cognitive behavioral theory (CBT) states that irrational thinking causes procrastination. Therefore, in the context of dealing with academic procrastination, CBT emphasizes efforts to change beliefs and irrational thoughts to rational thoughts. Zhang et al. (2022) found that negative and irrational thinking leads to academic procrastination. One of the factors influencing negative thinking includes an intervention program that is less effective and does not represent the entire domain, which is cognitive and behavioral (Toker and Avcı, 2015). The approach to overcoming academic procrastination is also deemed obsolete. Particularly, in Malaysia, the approach is only focused on counseling and motivational workshops for students to create positive thinking. However, such an approach is deemed less effective because it only focuses on the emotional aspect. The cognitive behavioral theory is also less commonly used in the context of education in Malaysia, unlike Turkiye, which has created many intervention programs such as psycho-education programs that use a cognitive-behavioral approach to overcome academic procrastination (Toker and Avcı, 2015). Essentially, the CBT program is believed to improve students’ basic skills to control cognitive and behavioral academic procrastination.

Therefore, a study related to the influence of learning styles on academic procrastination in the subject of Mathematics must be carried out to examine this issue among secondary school students in detail. Accordingly, the following hypotheses were proposed in the current study:

*H_0_*1: There is no significant influence of visual learning style on the academic procrastination of secondary school students in the subject of Mathematics.

*H_0_*2: There is no significant influence of auditory learning style on the academic procrastination of secondary school students in the subject of Mathematics.

*H_0_*3: There is no significant influence of kinesthetic learning style on the academic procrastination of secondary school students in the subject of Mathematics.

## 3. Methodology

### 3.1. Research design and sample

A quantitative approach with a survey research design was used in this study. Survey methods are appropriate based on the objectives of the study and coincide with the goals of quantitative research to make predictions between variables (Chua, 2021). The quantitative approach was used to determine the influence of learning styles on students’ academic procrastination in Mathematics. The survey research design is also capable of obtaining data from a large population (Azizi et al., 2021). The research population includes Form Two and Form Four secondary school students at National Secondary Schools in Kota Bharu district, Kelantan. The total student population in this study was 10,071, while the sample involves 500 respondents. Therefore, the survey method is most appropriate, especially since the study involves a large population (Chua, 2021). In addition, the survey method was chosen because information can be gathered in a short time (Darusalam and Hussin, 2021). This will make the data and information easier to analyze, in addition to saving time.

The respondents of this study were selected through simple random sampling so that each respondent in the population has an equal chance of being selected as a respondent (Chua, 2021). This study involved Form Two and Form Four secondary school students at National Secondary Schools in Kota Bharu district, Kelantan. The rationale for selecting Form Two and Form Four students is that these students are considered mature (Ahmad and Ambotang, 2020) enough to provide rational feedback or answers and can better understand the instructions given in the questionnaire. In addition, Form Two students were selected because they would sit for special assessments such as entrance tests to specialized schools, while Form Four students would sit for the Malaysian Certificate of Education (SPM) examination next year (Afzan and Abd Rahman, 2021). In terms of the selection of the research location, Kelantan was chosen because the state showed an increase in the achievement of the Malaysian Certificate of Education (SPM) 2021. Furthermore, Harian Metro also reported 100% achievement of passing SPM 2021 in 31 schools in Kelantan (Idris, 2022). This shows that students in the state of Kelantan have high enthusiasm and motivation in their learning. The duration of data gathering started from 4 October 2022 until 31 January 2023.

### 3.2. Questionnaire instrument

The questionnaire instrument in this study was adapted from Mamickam (2010) and Ghazal (2012). The three main sections of the questionnaire include demographics (3 items), students’ learning styles (31 items), and academic procrastination (21 items). The instrument was adapted and translated into Malay to meet the context of mathematics learning. Specifically, the questionnaire was translated to meet the values and norms of the local community as well as to determine the validity and reliability of the items in the questionnaire (Yusoff et al., 2018). The demographic section includes the respondents’ backgrounds such as age, gender, and socioeconomic status. Sections related to students’ learning styles were categorized into three modalities, namely visual, auditory, and kinesthetic, where visual and auditory modalities comprise 11 items, respectively, followed by kinesthetic with 9 items.

A three-point Likert scale was used (1 = rarely practiced, 2 = sometimes practiced, 3 = often practiced). The use of the three-point Likert scale is in line with the aim to identify students’ learning styles in Mathematics. Meanwhile, the items for academic procrastination are based on a 5-point Likert scale (1 = strongly disagree, 5 = strongly agree). The five-point Likert scale was used because the reliability coefficient increases with the increasing number of response options (Taherdoost, 2019). The mean score interpretation for the five-point Likert followed the Zaidatol (2009) that more than 3.80 (as high), range 3.40 to 3.79 (as moderate), and less than 3.39 (as low). The 5-point scale is also easy to understand and the respondents can express their views freely owing to the neutral option provided (Park et al., 2021). This will increase the response rate from respondents.

### 3.3. Validity and reliability

Eight experts in the field of psychology and counseling were appointed to verify the content of the instrument, which coincides with Lynn (1986) who advocated a minimum of three experts. Feedback from experts was used as a reference to improve the research instrument in terms of language and appropriateness of sentences to suit the context of the study and students in Malaysia. Content validity analysis was carried out using content validity ratio (CVR) analysis. Specifically, CVR was chosen to measure the content validity of items because it is more user-friendly and transparent and has been widely used by researchers. CVR is also easier to use because it involves simple calculations and the determination of the critical cut-off value is also available (Lindell and Brandt, 1999).

Before the content validity process was carried out, initially, the total number of items used in the instrument was 51, i.e., 30 items for learning styles and 21 items for academic procrastination. In general, the expert panel certified that the items could measure the content of each construct; however, some issues were presented by some experts to improve the content of the items in the instrument. Based on the calculation of CVR values per the evaluation of the expert panel, 15 items recorded a CVR value less than the critical level of 0.75. Table 1 shows the distribution of CVR values for all items according to each variable in detail. Eight items in the learning style variable did not reach the required cut-off level, while seven items in the academic procrastination variable must be re-evaluated.

**Table 1:** CVR value distribution of items.

CVR	Number of items for learning styles	Number of items for academic procrastination	Action
1.00	9	2	Accepted
0.75	13	12	Accepted
<0.75	8	7	Re-evaluated

For the 15 items that did not meet the CVR critical value, experts raised several issues such as item clarity (8 items), repetitive items (4 items), and layered items (3 items). Based on expert comments and suggestions, some items with issues from the aspect of item clarity, repeated items, and layered items were improved and refined to further enhance the quality of the instrument. One of the refined items was also divided into two separate items per the experts’ recommendations. Therefore, following a total of 51 initial items that were distributed to experts for content validity, there were 52 final items consisting of 31 items learning style variable including (11 items for visual and auditory each, with 9 items for kinesthetic) and 21 items for academic procrastination.

A pilot study was conducted in a secondary school in Bachok district, Kelantan, involving 60 students consisting of Form Two and Form Four students. Table 2 shows the reliability test results based on constructs, where Cronbach’s alpha coefficient value for the entire learning style constructs was 0.737. Cronbach’s alpha coefficient value for the visual modality was 0.677, followed by auditory with 0.573 and kinesthetic with 0.583. Academic procrastination overall showed Cronbach’s alpha coefficient of 0.905. This value is more than 0.7 and consider as a valid, good and acceptable value (Sekaran and Bougie, 2016).

**Table 2:** Cronbach’s alpha coefficient value analysis for each construct.

Construct	Cronbach’s alpha	Number of items
Learning style	0.795	31
Learning style - Visual	0.677	11
Learning style - Auditory	0.573	11
Learning style - Kinesthetic	0.583	9
Academic procrastination	0.907	21

A total of 600 copies of the questionnaire were distributed to the five schools involved; however, only 500 sets of questionnaires had complete data for analysis. The collected data were then analyzed using Statistical Package for Social Sciences (SPSS) software version 26.0. Descriptive analysis was used to identify the learning styles of students in the subject of Mathematics and their level of academic procrastination in the subject, while the multiple regression analysis determined the influence of learning styles on the academic procrastination of Form Two and Form Four students in Mathematics.

## 4. Research findings

The objectives of this study are (a) to identify the learning styles of students in the subject of Mathematics in secondary schools, (b) to identify the level of students’ academic procrastination in the subject of Mathematics, and (c) to determine whether there is a significant influence of learning styles (visual, auditory, and kinesthetic) on the academic procrastination of secondary school students in Mathematics.

The discussion of the descriptive analysis results was based on the first and second objectives. The results for the first objective were obtained by analyzing the overall scale score based on scales of 1 = rarely practiced, 2 = sometimes practiced, and 3 = often practiced. Data for each item was analyzed based on frequency (*f*), percentage (%), and median. Next, the overall scale score for each construct, i.e., visual, auditory, and kinesthetic, was analyzed using mean values and standard deviation. The analysis of each learning style construct in the subject of Mathematics is shown in Table 3.

**Table 3:** Analysis of learning style constructs.

Learning style construct	Mean	Standard deviation
Visual	23.89	3.83
Auditory	22.87	3.44
Kinesthetic	16.66	3.15

Overall, based on the mean scores, the visual learning style was the most dominant among Form Two and Form Four students in Mathematics, followed by auditory and kinesthetic learning styles.

The next analysis results were based on the second objective, which is to identify the level of students’ academic procrastination in the subject of Mathematics. Items in the academic procrastination construct were measured using a 5-point Likert scale. The overall scale score for academic procrastination was also analyzed based on the mean value and standard deviation. Table 4 shows the analysis results for the construct of academic procrastination. Evidently, the findings showed that the level of students’ academic procrastination in the subject of Mathematics was low.

**Table 4:** Analysis of academic procrastination construct.

Academic procrastination construct	Mean	Standard deviation
Academic procrastination	3.04	0.55

Finally, the inferential analysis results were based on the third research objective. A multiple regression analysis was carried out to determine the influence of learning styles on students’ academic procrastination in the subject of Mathematics. Assumptions for the use of multiple regression analysis have been met, including data measured on an interval scale, normal data distribution based on the normality test through skewness and kurtosis values, homoscedasticity, and the existence of a linear relationship between variables. Tables 5–7 show the multiple regression analysis results.

**Table 5:** Regression model summary.

Model	*R*	*R^2^*	Adjusted *R^2^*
1	0.376	0.141	0.136

**Table 6:** Variance analysis of regression model.

	Sum of squares	*df*	Mean squared	*F*	*p*-value
Regression	21.141	3	7.047	27.167	0.000
Error	128.663	496	0.259		
Total	149.803	499			

**Table 7:** Multiple regression coefficient analysis.

Variable	Unstandardized coefficient *B*	Unstandardized coefficient *SE*	Standardized coefficient beta	*t*	*p*-value
Constant	2.164	0.175		12.335	0.000
Visual	0.062	0.007	0.433	8.621	0.000
Auditory	−0.011	0.008	−0.068	−1.314	0.189
Kinesthetic	−0.021	0.008	−0.123	−2.576	0.010

Overall, based on the analysis, the regression model was significant [*F*(3,496) = 27.17, *p* < 0.001, and *R*^2^ = 0.141]. Visual (*b* = 0.062, *t* = 8.621, *p* < 0.001) and kinesthetic (*b* = −0.021, *t* = −2.576, *p* < 0.001) learning styles were significant predictors of academic procrastination. The analysis also showed that learning styles could explain 14.1% of the variation in academic procrastination (*R*^2^ = 0.141), while the remaining 85.9% were explained by other factors that were not examined in this study.

Analysis of the Beta standardized coefficient value in Table 7 showed that the standardized coefficient value for the visual learning style (0.433) was greater than that of the kinesthetic learning style (−0.123). The null hypothesis was successfully rejected. There was a significant influence of visual and kinesthetic learning styles on students’ academic procrastination in the subject of Mathematics. The findings also showed that the visual learning style had a greater effect on students’ academic procrastination in the subject of Mathematics compared to the kinesthetic learning style.

Overall, the contribution of learning styles to the variance in students’ academic procrastination in the subject of Mathematics can be formed through a regression equation model, as follows:


$$\begin{array}{c} Academicprocrastination=2.164+\left(0.062\right)\;Visuallearningstyle\\ +\;\left(-0.021\right)\;Kinestheticlearningstyle \end{array}$$

Based on the above equation, for every increase of one unit in visual learning style, students’ academic procrastination in the subject of Mathematics would increase by 0.062 units. Meanwhile, for every increase of one unit in kinesthetic learning style, students’ academic procrastination in the subject of Mathematics would decrease by 0.021 units.

## 5. Discussions

### 5.1. Objective 1: to identify students’ learning styles in the subject of mathematics in secondary schools

The first research objective is about the learning styles of students in the subject of Mathematics in secondary schools. Overall, the findings showed that the majority of Form Two and Form Four students practiced the visual learning style, followed by auditory and kinesthetic learning styles. This agrees with Nithya Dewi et al. (2019) who examined student learning styles in Mathematics in which different learning styles were identified according to the students’ inclinations. Although there were differences in student learning styles in the subject of Mathematics, Nithya Dewi et al. (2019) in their study showed that the majority of the students adopted a visual learning style when learning mathematics. This study was supported by Apipah (2018) as well as Savitri and Rochmad. (2022) who found that students typically begin to build an understanding of mathematical concepts through visuals. Moreover, the same findings were also reported by Ahmad et al. (2021) who conducted a study on Form Five students in a cluster secondary school in Pahang. In essence, all the above past findings have strengthened the findings of the current study on the learning styles of students in the subject of Mathematics.

To master mathematical concepts well, critical thinking in mathematics is essential. Ahmad et al. (2021) stated that the right learning style helps sharpen students’ critical thinking in the subject of Mathematics. Critical thinking in mathematics learning requires a high level of cognitive skills, and solving mathematical problems involves critical thinking skills. Therefore, students need to know how to solve mathematical problems by applying the correct formulas and concepts as well as explaining concepts and finding solutions to mathematical problems. Accordingly, when students adopt an appropriate learning style, they can improve their critical thinking skills in the subject of Mathematics.

Better mastery of mathematical concepts was demonstrated by students with a visual learning style because Mathematics is closely related to formulas, graphs, and diagrams. Students with a visual learning style visualize these images to ensure that the mathematical knowledge acquired can be stored in long-term memory through careful observation (Apipah, 2018). As such, mathematics learning requires strong visual skills in interpreting information so that students can master mathematical concepts better. Apipah (2018) also explained that the visual learning style helps students to make good mathematical connections—a skill that helps students to connect each mathematical idea and makes it easier for them to summarize each mathematical concept. As a result, students can improve their understanding of the mathematical concepts learned in class. Choosing the wrong learning style makes it difficult for students to understand the relevance of mathematics. This is because, through learning styles, the process of learning mathematics is absorbed, processed, and developed more deeply.

Based on the findings reported by Apipah (2018), students who practice the visual learning style record the steps to solve mathematical problems in a systematic, organized, and clear manner. This is because visual students are more likely to visualize or imagine the steps in a mathematical solution before attempting to solve a mathematical problem. Thus, the findings of the study coincide with the characteristics of the visual learning style where visual students learn better by looking at diagrams or imagining concepts or formulas before translating them into real mathematical problem-solving.

In addition, the findings of the study showed that the auditory learning style was the second-highest learning style in the subject of Mathematics. This is different from the study conducted by Virgana (2019) where the auditory learning style was the dominant learning style in this subject. However, in this study, the levels of visual and auditory learning styles did not show significant differences. This is closely related to the teacher’s teaching method in the classroom, which is still contingent on the traditional teaching style where the teacher merely explains topics and mathematical concepts in the classroom. Furthermore, the teaching method of using explanations as the main source of mathematics learning also causes students to practice auditory learning style in the subject of Mathematics.

Meanwhile, the kinesthetic learning style recorded the lowest level in Mathematics, and this finding is supported by Savitri and Rochmad. (2022) who stated that kinesthetic students find it difficult to make mathematical connections well because they learn by manipulating objects or applying them to everyday life. Briefly, since mathematics learning is more abstract (Hui and Rosli, 2021), it is difficult for these students to relate the mathematical concepts learned to their real life. Furthermore, the teaching method of Mathematics teachers, which is entirely contingent on explanations in class without diversifying the teaching methods, limits kinesthetic students’ understanding of abstract mathematical concepts. Students with a kinesthetic learning style also learn to understand mathematical concepts with body movements such as moving fingers or hands. This makes it difficult for them to maintain focus or concentration in order to understand mathematical concepts well (Sulisawati et al., 2019).

Mathematics learning, which is abstract, makes it difficult for students to process mathematical content without guidance and explanation from the teacher. According to Pramudya et al. (2021), due to mathematical concepts that are too abstract, most teachers tend to teach students by explaining mathematical concepts in class and then reinforcing students’ understanding with mathematical exercises. However, students tend to experience difficulties in understanding the terms or terminology and instructions for solving mathematical problems (Sarudin et al., 2019). Consequently, the problems encountered by the students make it difficult for them to master mathematical concepts well.

This situation further causes mathematics learning in the classroom to become passive. Thus, to create an active mathematics learning process, teachers need to act as facilitators in guiding the students’ mathematics learning process. However, in Malaysia, teachers serve as the main source or driver in the delivery of mathematics content. Students’ self-learning, which has yet to reach a satisfactory level, also makes it difficult for teachers to change their teaching methods to one that is more student-centered (Pramudya et al., 2021). Thus, the findings of past studies are in line with the current findings, which highlighted the learning styles of students in the subject of Mathematics. Evidently, students preferred to practice visual and auditory learning styles when learning mathematics.

From a theoretical point of view, some students have good cognitive skills owing to effective learning style factors. By integrating cognitive behavioral theory (CBT) into mathematics learning, students are able to think positively by adopting an appropriate learning style based on their inclinations. The right learning style will reduce negative emotions in mathematics learning such as anxiety as well as low levels of motivation and self-efficacy in Mathematics. Students with high mathematics anxiety have difficulty controlling their negative emotions. Thus, several studies have used cognitive behavioral interventions to reduce students’ mathematics anxiety, such as by changing the way students think (Asanjarani and Zarebahramabadi, 2021). Mathematics anxiety typically occurs due to the way mathematical content is introduced to students and it does not necessarily stem from students’ difficulties in mastering the mathematical content itself (Samuel and Warner, 2021).

In this regard, it is very important for teachers to change students’ thinking and steer it toward a more positive direction by helping them plan effective strategies when learning mathematics. One of the effective mathematics learning strategies involves practicing an effective learning style that is compatible with students’ inclinations so that they can receive and process mathematical knowledge; students will also feel more confident when they can master mathematical concepts and skills well. As a result, a positive and active mathematics learning atmosphere among students will be realized.

### 5.2. Objective 2: to identify the level of students’ academic procrastination in the subject of mathematics

The second research objective highlights the level of students’ academic procrastination in the subject of Mathematics. The findings showed that students’ academic procrastination in Mathematics was at a low level, indicating that students had good study practices in the subject of Mathematics.

In general, the low level of students’ academic procrastination in the subject of Mathematics reflects the students’ positive spirit in learning this subject. Students also had a good attitude toward their learning time to ensure that every mathematics assignment can be completed on time. Good time management helps students plan their learning more efficiently so that every task given by the teacher can be completed perfectly (Ramadhan et al., 2021). Besides, the low level of academic procrastination in the subject of Mathematics suggests that students were aware of every instruction and mathematics assignment given to them, and they also had a high awareness of the importance of a mathematical task or exercise in order to improve their academic performance in the subject of Mathematics.

The positive behavior of students, such as not delaying mathematics assignments, also reflects their high motivation to deepen and learn mathematical topics seriously. Smart students take the opportunity to improve their understanding of mathematical concepts through mathematical exercises given by teachers. As such, when learning mathematics, students do not consider difficult mathematical tasks an obstacle for them to complete the tasks. In fact, they see the difficulty as a challenge and motivation to better strengthen their understanding of mathematical concepts (Hasbullah, 2021; Jeremy et al., 2021). For example, when students are unable to solve certain mathematical problems on some practice questions, they can effectively identify their weaknesses in the mathematical topic.

Mathematical exercises with various difficulty levels also help students to identify their level of understanding of a mathematical topic, making it easier for them to focus on the topic. When students are able to complete mathematics assignments, they will feel more confident in the mathematical concepts learned. Consequently, negative emotions in mathematics learning can be overcome.

Moreover, the low level of academic procrastination in the subject of Mathematics among students helps teachers plan their learning strategies. Teachers are more enthusiastic to teach mathematical topics in class because students take every task entrusted to them seriously. Teachers can also reflect on teaching through the mathematics assignments given to students (Ariffin, 2017), besides identifying students’ weaknesses from the way they solve mathematical problems in a given task. Next, teachers can help students to focus more on questions that are considered difficult (Listiwikono, 2022). For instance, teachers can thoroughly and frequently explain the mistakes that students always make when solving mathematical problems based on their review of the students’ mathematics assignments.

In addition, teachers can also emphasize difficult mathematical topics by increasing the mathematical exercises to be done so that students’ understanding of the topics can be enhanced. This can be ensured when students are able to complete their mathematics assignments well because mathematical questions or exercises can be used as benchmarks for teachers to assess students’ understanding of the subject of Mathematics. Besides, due to the nature of mathematics learning, which mainly depends on the ability and skills of students to solve mathematical problems, students can be given many mathematical exercises to help build their understanding of the subject and strengthen mathematical concepts by applying the correct mathematical formulas to solve mathematical problems.

Since this study is only focused on secondary school students, the findings may differ from the previous studies focusing on students’ academic procrastination in the subject of Mathematics at the university level. Research on academic procrastination in Mathematics in Malaysia is also very limited compared to Indonesia. Based on the findings of previous studies, the level of students’ academic procrastination in the subject of Mathematics was high (Setiyowati et al., 2020; Agustin and Winarso, 2021). The findings of the current study are different, as a decrease in the level of students’ academic procrastination in the subject of Mathematics was observed, i.e., from a high level to a low level.

The significant difference in the level of students’ academic procrastination in the subject of Mathematics in this study may be due to the respondents and the research design used. Previous studies employed university students as respondents compared to the current study, which only involved secondary school students. In addition, this study only used a survey research design and involved a large number of respondents (i.e., 500) compared to the previous studies using interviews and observations as well as involving a small number of respondents. Although these factors may explain the difference in results, further research is still needed to strengthen and confirm the difference.

Furthermore, the difference in the level of students’ academic procrastination in the subject of Mathematics may be due to the positive changes taking place in the Malaysian education system. Various efforts and initiatives have been taken by the Ministry of Education Malaysia (MoE) to empower the country’s education, such as introducing a special Educational TV channel through KPM’s DidikTV to help students study at home and provide mathematics teaching and learning (PdP) support materials for the use of students and parents, which can be accessed online. The initiative taken by the MoE encourages continuous learning of mathematics. Students do not have to wait to acquire the full source of information through teachers at school; instead, students can practice self-learning when completing mathematics assignments to improve their understanding of mathematical concepts.

From a theoretical perspective, students’ academic procrastination is closely related to negative emotions and irrational thoughts that students face when learning mathematics (Hima and Sari, 2021), which affects their cognitive ability. Students who procrastinate on mathematics assignments are motivated by fear of failure or high mathematics anxiety. This happens because students are not confident in their abilities and mathematical skills; hence, they will try to avoid thinking about and doing mathematical tasks. In this vein, through the application of the cognitive behavioral theory, students are made aware of the importance of overcoming academic procrastination in the subject of Mathematics. In addition, effective cognitive behavior can also foster students’ positive thinking toward mathematics learning (Asanjarani and Zarebahramabadi, 2021). Essentially, positive thinking among students can make them more efficient and confident to learn mathematics and this further increases their efforts to improve their weaknesses in difficult mathematical concepts.

Overall, although the level of students’ academic procrastination in the subject of Mathematics was low, focus still needs to be given to ensure that mathematics achievement can be improved. The factors affecting students’ academic procrastination in the subject of Mathematics must also be examined in detail to determine the long-term effect of these factors on student learning, particularly in the subject of Mathematics so that measures can be taken to curb academic procrastination in Malaysia.

### 5.3. Objective 3: to determine whether there is a significant influence of learning styles (visual, auditory, and kinesthetic) on the academic procrastination of secondary school students in the subject of mathematics

The multiple regression analysis results showed that visual and kinesthetic learning styles had a significant influence on the academic procrastination of students in the subject of Mathematics. The positive beta standardized coefficient value indicates that students who adopted a visual learning style contributed to academic procrastination in the subject of Mathematics. Generally, visual students are more likely to learn with the help of texts or pictures and by looking at diagrams or real objects; therefore, when there are pictures or illustrations that are deemed more interesting than their reading materials at that time, visual students are more easily distracted by the attraction of colorful illustrations or diagrams found in other reading materials.

As a result, visual students tend to lose focus of their initial learning goals easily. This is supported by Mahadi et al. (2022) who stated students with a visual learning style are more easily distracted than those with other learning styles. When visual students find it difficult to fully focus on mathematics learning in class, they cannot understand the mathematical concepts being presented and this will lead to academic procrastination because they cannot apply mathematical concepts in mathematical tasks.

Since kinesthetic students also had a significant influence on academic procrastination in the subject of Mathematics, the negative beta standardized coefficient value suggests that students’ academic procrastination in Mathematics decreased with kinesthetic learning style. This is probably because kinesthetic students use many hands-on or applications with real situations, which increases their interest in learning mathematics. Students with a kinesthetic learning style are also more confident in their learning when touching objects or engaging in hands-on learning activities (Mahadi et al., 2022). For example, in the topic of polygons, kinesthetic students can better understand the concept of polygons when they relate the combinations of polygon shapes around them. A good student’s mastery of mathematical concepts is demonstrated through mathematical exercises; when students can complete mathematics assignments perfectly, academic procrastination can be overcome.

The third objective finding supports the flow of theoretical supports of ideas from VAK learning styles model and cognitive behavioral theory. Although the influence of learning styles on academic procrastination among students in mathematics is only 14 percent, this finding shows the existence of the interaction of emotional, cognitive and behavioral components that result in the practice of student academic procrastination in the subject of Mathematics. In this research context, cognitive behavioral theory provides the students with a way of understanding to the world experience or mathematics learning, and enabling students to make changes if they need to. It does this by dividing the students experience into central components: namely thoughts (cognitions), feelings (emotions), and behaviors except for physiology (biology) that has not been discussed in this paper. All these elements show the interaction of students’ irrational thinking, unstable emotions and less efficient learning styles causing academic procrastination in Mathematics.

Through cognitive behavioral theory, students’ knowledge in Mathematics can be built through positive thinking and behavior. Cognitive behavioral theory states that irrational thinking causes procrastination. In the context of dealing with academic procrastination, CBT emphasizes efforts to change beliefs and irrational thoughts to rational thoughts. Zhang et al. (2022) found that negative and irrational thinking leads to academic procrastination. One of the factors influencing negative thinking includes an intervention program that is less effective and does not represent the entire domain, which is cognitive and behavioral (Toker and Avcı, 2015).

Cognitive behavioral specifically is a form of psychological part that has been demonstrated to be effective for a range of problems including depression, anxiety disorders, alcohol and drug use problems, marital problems, eating disorders, and severe mental illness. The students involved in this study are Form Two and Form Four students in secondary schools among 14 to 16 years face many challenges including in learning Mathematics. In fact, students are likely to have some characteristics of depression, anxiety disorders, eating disorders, and severe mental illness as a result of stress in learning mathematics which can cause academic procrastination.

This finding also supported by VAK learning styles model by proving that visual and kinesthetic learning styles have a significant influence on students’ academic procrastination in Mathematics. Visual learning style is the most dominant learning style. Visual in this research highlighted on learners respond to images and graphics in learning Mathematics. Students begin to build an understanding of Mathematical concepts through visuals. Mastery of Mathematics concepts is better demonstrated by students with a visual style because Mathematics is closely related to formulas, formulas, graphs and diagrams. The visual learning style shows the ability to visualize images to ensure that the Mathematical knowledge gained can be stored for long-term memory through careful observation (Apipah, 2018). This shows that mathematics learning uses a lot of strong visual skills in interpreting information so that students can master Math concepts better. To master the concept of Mathematics well, critical thinking in Mathematics is really necessary.

In this context, respondents with a visual learning style indirectly show the need for cognitive behavioral theory. Apart of visual style requires long-term memory and strong visual skills, students have the potential to feel a little pressure and depression in learning mathematics which can cause academic procrastination. Students tend to not perform very well in their Mathematics assignments. Due to this, they may face psychological distress and thinks that they would fail the assignment. This is distorted thinking because there is also a chance that they would perform better in future. In this regard, the immediate action that need to be taken is improving the visual learning styles techniques for overcoming students’ academic procrastination in Mathematics.

## 6. Implication

As for the implications, this study suggests improvements in excellence programs in schools by giving exposure to students on how to identify the appropriate learning styles so that they can practice effective learning styles in the subject of Mathematics and overcome academic procrastination.

## 7. Limitation and future research

This study has certain limitations as it only focuses on Form Two and Form Four students in learning Mathematics; therefore, the findings of the study cannot be generalized to other subjects at school. This study is also limited to one independent variable, which is the learning styles of students toward their academic procrastination in Mathematics. The findings have shown that the learning style factor could only explain 14.1% of the total variance in students’ academic procrastination in the subject of Mathematics. This shows that there are other factors that can still influence students’ academic procrastination in the subject of Mathematics, which have yet to be examined. Future research can be carried out by identifying other factors that lead to academic procrastination in the subject of Mathematics in order to increase the motivation and self-efficacy of students in this subject.

## 8. Conclusion

Different learning styles of students have been identified based on their inclinations in learning Mathematics. There are three main types of learning styles practiced by students in the subject of Mathematics, namely visual, auditory, and kinesthetic learning styles. Visual learning style was the dominant learning style among students in the subject of Mathematics, followed by auditory and kinesthetic learning styles. The level of students’ academic procrastination in Mathematics was also low. In addition, the study found that visual and kinesthetic learning styles had a significant influence on students’ academic procrastination in the subject of Mathematics. Therefore, exposure to the different learning styles must be improved through excellence programs in schools to provide knowledge to students in order to allow them to identify their learning styles and practice an effective learning style so that the quality of student learning in the subject of Mathematics can be improved.

## Data availability statement

The original contributions presented in the study are included in the article/supplementary material, further inquiries can be directed to the corresponding author.

## Ethics statement

The studies involving humans were approved by Ministry of Education (MoE), Malaysia. The studies were conducted in accordance with the local legislation and institutional requirements. Written informed consent for participation was not required from the participants or the participants’ legal guardians/next of kin in accordance with the national legislation and institutional requirements.

## Author contributions

WW was involved in the collection of data, data analysis, and producing the first draft of this study. MM was responsible for reviewing and editing, as well as supervising. All authors are responsible for the concept and design of the study. All authors contributed to the article and approved the submitted version.
